# The Neuroprotective Marine Compound Psammaplysene A Binds the RNA-Binding Protein HNRNPK

**DOI:** 10.3390/md15080246

**Published:** 2017-08-07

**Authors:** Marco Boccitto, Nayoung Lee, Satoshi Sakamoto, Lynn A. Spruce, Hiroshi Handa, Jon Clardy, Steven H. Seeholzer, Robert G. Kalb

**Affiliations:** 1 Department of Pediatrics, Division of Neurology, Research Institute, Children’s Hospital of Philadelphia, Room 814, 3615 Civic Center Boulevard, Philadelphia, PA 19104, USA; marco.boccitto@nih.gov; 2 Department of Neurology, Perelman School of Medicine, University of Pennsylvania, Philadelphia, PA 19104, USA; 3 Department of Neuroscience, Perelman School of Medicine, University of Pennsylvania, Philadelphia, PA 19104, USA; 4 Department of Biological Chemistry and Molecular Pharmacology, Harvard Medical School, 240 Longwood Avenue, Boston, MA 02115, USA; nayoung.lee@genex.co.kr (N.L.); jon_clardy@hms.harvard.edu (J.C.); 5 School of Life Science and Technology, Tokyo Institute of Technology, 4259 Nagatsuta-cho, Midori-ku, Yokohama, Kanagawa 226-8501, Japan; ssakamoto@bio.titech.ac.jp; 6 Department of Nanoparticle Translational Research, Tokyo Medical University 6-1-1, Shinjuku, Shinjuku-ku, Tokyo 160-8402, Japan; hhanda@tokyo-med.ac.jp; 7 Children’s Hospital of Philadelphia Research Institute, Protein and Proteomics Core, Philadelphia, PA 19104, USA; spruce@email.chop.edu (L.A.S.); seeholzer@email.chop.edu (S.H.S.)

**Keywords:** Psammaplysene A, neurodegeneration, foxo, HNRNPK, RNA metabolism

## Abstract

In previous work, we characterized the strong neuroprotective properties of the marine compound Psammaplysene A (PA) in in vitro and in vivo models of neurodegeneration. Based on its strong neuroprotective activity, the current work attempts to identify the physical target of PA to gain mechanistic insight into its molecular action. Two distinct methods, used in parallel, to purify protein-binding partners of PA led to the identification of HNRNPK as a direct target of PA. Based on surface plasmon resonance, we find that the binding of PA to HNRNPK is RNA-dependent. These findings suggest a role for HNRNPK-dependent processes in neurodegeneration/neuroprotection, and warrant further study of HNRNPK in this context.

## 1. Introduction

Psammaplysene A (PA) is a marine sponge (*Psammaplysilla* sp.) metabolite which was originally described in a chemical screen to identify agents that promote FOXO1 nuclear localization in phosphatidylinositol 3,4,5-triphosphate 3-phosphatase-deficient cancer cells [1]. We became interested in the potential neuroprotective qualities of PA based on the role of the FOXO transcription factors as phylogenetically conserved regulators of longevity [2]. Increased activity of the *Caenorhabditis elegans* ortholog of FOXO3, *daf-16*, is associated with lifespan extension and increased resistance to stressors such as reactive oxygen species, heat and proteotoxicity [3,4,5]. Based on these observations, we investigated the ability of PA to act as a neuroprotective agent, potentially via activation of FOXO3. Indeed, we found that PA protects against neuronal death in numerous in vitro and in vivo models of neurodegeneration [6]. In mixed spinal cord cultures, PA was found to protect against neuronal death induced by excitotoxicity, as well as proteotoxicity evoked by expression of mutant superoxide dismutase (SOD1), mutant p150^glued^ and poly-glutamine expanded androgen receptor. PA was also protective against excitotoxicity in *C. elegans* and a *Drosophila* model of spinal-bulbar muscular atrophy.

Identification of the direct physical target of PA is likely to provide insight into the molecular mechanism of its neuroprotective qualities. To achieve this goal, we used two distinct strategies to identify protein interactors, and generated a list of candidates that consisted almost exclusively of RNA-binding proteins. From this list, we identified the target of PA as heterogeneous nuclear ribonucleoprotein K (HNRNPK). HNRNPK is a hub protein controlling many aspects of RNA biology as well as acting as an integrator of protein-based signaling cascades. Our results suggest that the neuroprotective actions of PA may be mediated by modifying HNRNPK-dependent process(es).

## 2. Results

### 2.1. PA Is Not “Drug-Like”

**Figure 1:**
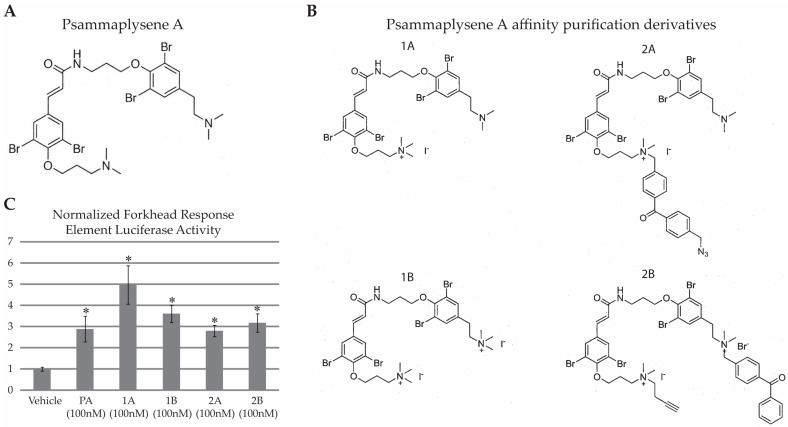
(**A**) The marine sponge compound Psammaplysene A (PA); (**B**) Four PA derivatives were synthesized to investigate the target of PA and (**C**) The four PA derivatives were screened for the ability to increase expression of a luciferase reporter which is enhanced by nuclear localization of FOXO transcription factors (* *p* < 0.05).

Chris Lipinski and colleagues defined a set of four characteristics that suggest whether a molecule is “drug-like”; (1) molecular weight (≤500 Da); (2) Log *P* (a measure of lipophilicity, ≤5); (3) number of hydrogen bond donors (≤5) and (4) number of hydrogen bond acceptors (≤10) [7]. Each time a compound violates one of these criteria, it is given a “flag” and the total number of flags are tallied, giving a compound a score between 0 and 4. Compounds with a score greater than 1 are considered to be of marginal value for further development [8]. PA (Figure 1A) receives a score of 2 for having a molecular weight of 769 Da and a Log *P* value of 6.57, suggesting it would be of marginal utility as a drug. Our original characterization of PA as neuroprotective took advantage of the fact that testing in vitro and in small model organisms such as *C. elegans* and *Drosophila* allows for the use of DMSO for drug solubilization and delivery. Owing to PA’s neuroprotective qualities [6], we wanted to characterize its target, with the goal of identifying a protein that is potentially a modifier of neurodegeneration.

### 2.2. HNRNPK Is a Target of PA Based on Two Parallel Target Purification Strategies

Two separate but complementary approaches were used to purify the target of PA from cell lysates. The first approach involved a modified version of PA that allowed for covalent linkage of PA to interacting protein(s) and tagging with a biotin moiety or fluorescent probe. The second approach involved covalently linking PA to magnetic nanobeads using a flexible linker sequence.

#### 2.2.1. Purification of Proteins Associated with a Photo-Crosslinkable PA Derivative

Four different PA derivatives were synthesized (Figure 1B). To determine if these modifications altered PA activity, we compared the activity of the four PA derivatives to PA in a forkhead response element (FHRE) luciferase assay (Figure 1C). We had previously shown that PA increases FHRE luciferase activity, and suggested that its neuroprotective properties are likely associated with this ability to influence FOXO activity [6]. We found that PA, as well as all four PA derivatives, elicited a statistically significant increase in FHRE activity compared to vehicle (single factor ANOVA *F*_(5,66)_ = 7.09, *p* < 0.01). Post hoc analysis showed vehicle-treated cells to have significantly less FHRE activity compared to all other groups (*p* < 0.05 when comparing vehicle- vs. all drug-treated groups by Tukey’s post hoc). Since the derivative compounds retained the ability to enhance FOXO-dependent transcription, we presumed that they were still capable of binding the bona fide target of PA. The photoactivatable crosslinker and azide groups on compound 2B provided a versatile platform to work with, and therefore we conducted subsequent derivative-based purification assays with compound 2B.

**Figure 2:**
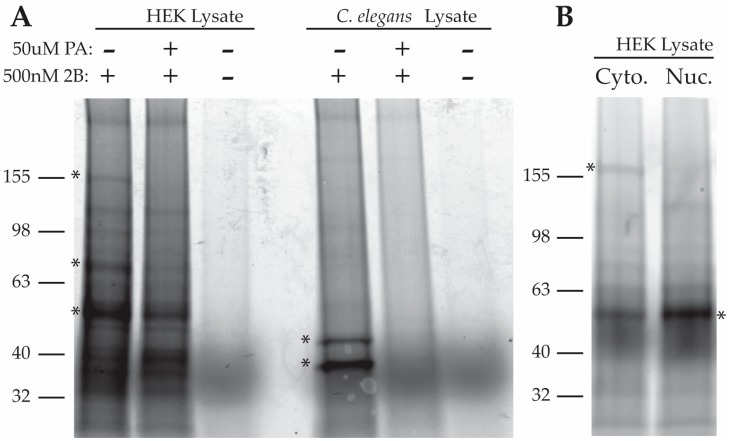
(**A**) Visualization of 2B bound proteins in HEK293 cell and *C. elegans* lysates. * indicate bands that were diminished by the addition of PA; (**B**) Nuclear and cytoplasmic distribution of 2B target proteins in HEK293 cell lysates.

We incubated HEK293 and *C. elegans* lysates with 2B, 2B + 100x PA, or no drug. After a one-hour incubation, we UV-illuminated the lysates to crosslink 2B to its target and then used Click chemistry to label 2B-containing complexes with carboxytetramethylrhodamine (TAMRA). After labeling, proteins were dissolved in loading buffer, resolved by SDS-PAGE and TAMRA-labeled proteins were visualized using in-gel fluorescence scanning (Figure 2A). Three specific bands were identified in HEK293 lysates with approximate molecular weights of 160 kDa, 75 kDa and 50 kDa. A similar result was found when using rat neocortical neuron lysates (data not shown). Since neuronal samples appeared to contain the same targets as HEK293 cells, we carried out all further target purifications with HEK293 cells as this allowed us to use greater quantities of input protein. Since we had also previously found PA to be neuroprotective in *C. elegans* models of neurodegeneration, we performed the 2B crosslinking assay in *C. elegans* lysates. Two specific bands were identified in *C. elegans* lysates with approximate molecular weights of 40 kDa and 45 kDa (marked with *). Compound 2B binding appeared specific since its interaction with the above proteins was compromised or abolished in the presence of excess of PA (Figure 2A). Proteinase K pretreatment of samples eliminated these bands suggesting that they are proteins (data not shown). The fact that 2B appears to bind specific protein targets in both mammalian cells and *C. elegans*, two systems where we found PA to be neuroprotective, enhanced our confidence that PA’s mechanism of action is mediated by binding to a discrete protein target rather than a non-specific or non-protein-based mechanism of action. Subcellular fractionation revealed an exclusively cytoplasmic localization of the 160 kDa band (upper *) while the 50 kDa band (lower *) appeared in both cytoplasmic and nuclear fractions, with enrichment in the nuclear fraction (Figure 2B). The 75 kDa band was difficult to follow in this assay.

Next, in order to identify the 2B-interacting proteins in the HEK293 lysate, we incubated 2B with HEK293 cell lysate, UV-crosslinked, used Click chemistry to attach a biotin to crosslinked target-2B complexes and then purified target-2B-biotin complexes using avidin-coated magnetic beads. In an effort to increase the stringency of the assay, a series of biotin–avidin pulldown experiments were performed using buffers with various concentrations of salt, detergents and urea. We determined that the most stringent wash conditions that the biotin–avidin complex could withstand consisted of: 50 mM Tris pH 7.4, 1% Triton-X 100, 1M KCl, and 500 mM urea. Subsequently, the purified proteins were digested off of the avidin beads and analyzed by liquid chromatography–tandem mass spectrometry (LC–MS/MS). After excluding common contaminating proteins, we identified 31 candidate targets via this approach (Supplemental data S1_Samples_Report, 2B on Bead).

#### 2.2.2. Purification of PA-Interacting Proteins with PA-Coupled Nano Beads

**Figure 3:**
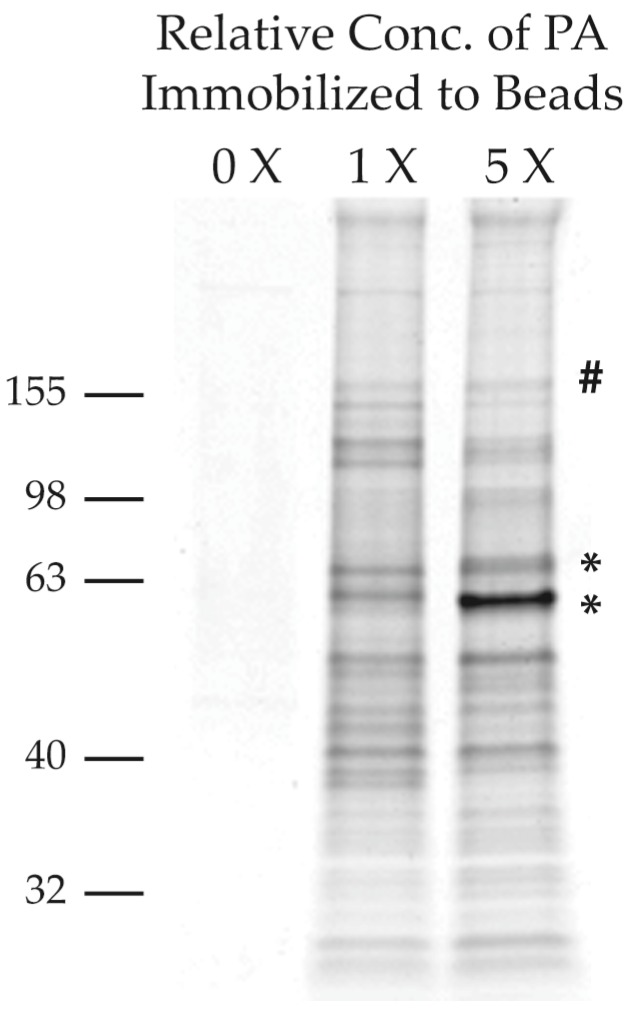
Visualization of PA-interacting proteins using PA-coupled nano-bead purification. Varying concentrations of PA were coupled to the FG nano-beads in order to identify proteins that showed a dose-dependent pulldown. Regions indicated by *’s and # were excised for LC–MS/MS analysis.

In a second, parallel, approach we covalently coupled a PA derivative to polymer-coated affinity magnetic (FG) nano-beads. These beads are particularly well suited for this type of analysis because their small size and uniform structure create a large surface area for small molecule presentation while minimizing non-specific interactions [9]. FG beads were coupled to PA at two different concentrations of PA, 1X and 5X. We incubated HEK293 lysates with uncoupled FG beads, 1X PA-coupled FG beads and 5X PA-coupled FG beads. Beads were washed, and then boiled in SDS loading buffer to elute the associated proteins. Eluted proteins were run on a SDS-PAGE gel and visualized via Coomassie Brilliant Blue staining (Figure 3). We assumed that bona fide PA-interacting proteins would increase in band intensity in a PA dose-dependent manner. We identified two bands whose intensity was strongly dose-dependent (Figure 3 *’s with approximate molecular weights of 50 kDa [bottom band] and 75 kDa [middle band]). These bands were excised and analyzed by LC–MS/MS after trypsin hydrolysis. One additional band was excised (# Figure 3) based on its molecular weight, roughly matching the molecular weight of the largest binding partner identified by the 2B approach (160 kDa [top band]). Since the PA-FG beads were not covalently linked to their target in this approach, we were unable to wash them as stringently as in the 2B approach (washes were performed with: 50 mM Tris pH 7.4, 150 mM NaCl, 1% Triton-X 100). Subsequently after excluding common contaminating proteins, we identified 163 proteins in the three excised bands (Supplemental data S1_Samples_Report, PA-FG Beads).

#### 2.2.3. Analysis of 2B and PA-FG Interacting Proteins

Based on our two parallel approaches, we identified a combined total of 184 candidate targets of PA. Using the photo-crosslinkable PA derivate 2B we identified 31 candidates, while the PA-coupled nano-beads yielded 163 candidates (S1_Samples_Report). Five keratins were found in both samples, but were excluded from our analysis as they are common contaminants as determined by the Common Repository of Adventitious Proteins (http://www.thegpm.org/crap/index.html). After these proteins were removed, seven candidate proteins were identified by both approaches: FUS, PABP, HNRNPH, HNRNPUL1, HNRNPK, RBM14, HSP70 (See S1_Samples_Report, 2B vs. PA-FG Beads for side-by-side comparison). Interestingly, none of the identified proteins were members of the FOXO family of transcription factors, nor were any of the well-characterized modifiers of FOXO activity present on our candidate list [10]. Despite not having a clear link to FOXO, there were functional similarities in the candidate proteins purified by the two approaches. A bioinformatics analysis of the purified proteins using the Database for Annotation, Visualization, and Integrated Discovery (DAVID v6.7) indicated that both approaches identified proteins which were highly enriched for RNA processing functions (Table 1). Six of the seven proteins identified by both approaches are RNA-binding proteins. This also potentially helped explain the large number of proteins that we purified, since RNA-processing proteins often function as part of large macromolecular complexes [11]. For example, a number of proteins on our list are components of the hnRNP and snRNP complexes. While the FG beads are designed to have very few non-specific interactions, owing to the less stringent washing conditions, it is likely that many native protein–protein interactions would be preserved.

#### 2.2.4. Screening of PA-Interacting Candidate Proteins

**Figure 4:**
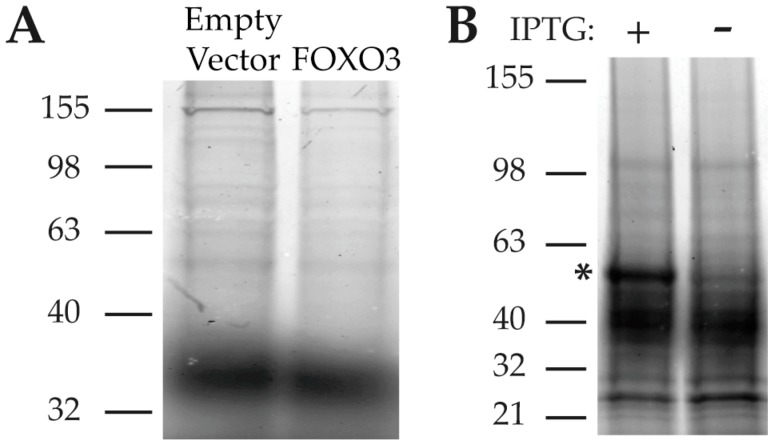
Targets identified by LC–MS/MS in both purification approaches were screened via the 2B binding assay in bacterial of mammalian lysates overexpressing the candidate target proteins. (**A**) Despite its effect on FOXO3 transcriptional activity, 2B does not interact with FOXO3; (**B**) 2B interacts with HNRNPK expressed in *E. coli*.

We obtained plasmids for recombinant protein over-expression or knockdown of candidate proteins and then screened cell lysates using the 2B TAMRA coupling assay. Fifteen proteins were screened in this manner: DDX3X, DDX5, DDX17, FUS, FOXO3, HNRNPH, HNRNPK, HNRNPL, HNRNPM, HNRNPR, HNRNPQ, HNRNPUL1, LA, RBP14, YTHDC2. In parallel, we also examined binding to FOXO3, despite the fact that FOXO3 was not identified by either target purification method, as previous work had identified a functional effect of PA on FOXO3. When we compared lysates from cells over expressing FOXO3 to cells expressing empty vector, we saw no difference in 2B labeling in these lysates (Figure 4A)—essentially excluding the possibility that FOXO3 is directly targeted by PA. After screening the fifteen targets from the candidate lists, we ultimately identified a positive hit when probing lysates from bacteria engineered to inducibly express HNRNPK. This yielded a strong 2B positive band at ~50 kDa molecular weight upon induction with IPTG (Figure 4B). The molecular weight of this band corresponds well with the band identified in HEK293 lysates.

Gene ontology analysis of the targets identified via the 2B purification and the PA-FG beads were consonant with the notion that the target of PA is HNRNPK. HNRNPK is an RNA-binding protein known to be involved in numerous aspects of RNA processing, including translation, mRNA stability, and splicing [12]. This makes HNRNPK a good fit for the types of processes that we suspect the target of PA is involved in based on our bioinformatics analysis of candidate proteins (Table 1). Subcellular fractionation followed by visualization of the target of PA, through fluorescent tagging of the 2B-target complex, showed that the 50 kDa target of PA is present in both the cytoplasm and the nucleus with an enrichment in the nuclear fraction (Figure 2B). This is consistent with the known subcellular distribution of HNRNPK (as well as our own observations by western (Supplemental Figure S1)). HNRNPK is a “hub” protein, interacting with numerous protein partners (>100) [13] and acting to integrate various signaling cascades. This breadth of protein–protein interactions is consistent with the high number of candidates identified by our purification methods. An analysis of the literature on HNRNPK-binding partners indicates that 12 of the 31 candidates (≈40%) identified by on-bead digest after 2B purification were previously described HNRNPK-binding partners. Taken together, these data support the idea that HNRNPK is a bona fide target of PA.

### 2.3. Characterization of the PA Interaction with HNRNPK by Surface Plasmon Resonance

**Figure 5:**
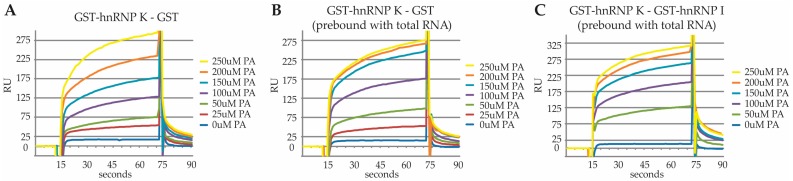
Surface plasmon resonance demonstrating saturable binding of PA to RNA-bound HNRNPK. (**A**) Real-time response of PA binding to HNRNPK-GST vs. GST in response units (RU) over the 0–250 µM range; (**B**) Real-time response of PA binding to RNA saturated HNRNPK-GST vs. GST in RU. (**C**) Real-time response of PA binding to RNA saturated HNRNPK-GST vs. RNA saturated HNRNPI-GST in RU. See S2_SPR_data for raw data.

We next characterized the binding of PA to HNRNPK by surface plasmon resonance. We began by comparing PA binding to GST-HNRNPK versus GST alone. We were unable to identify saturable binding in this paradigm (Figure 5A). We speculated that because HNRNPK participates in such a wide variety of protein-, RNA- and DNA-binding events, many aspects of its native structure may not be recapitulated during recombinant expression and purification. Although it would be impractical to try and introduce all of the potential HNRNPK-interacting proteins into the system, we wondered whether introduction of RNA would influence PA binding to HNRNPK in this assay.

We found that application of total RNA to immobilized GST-HNRNPK or GST led to saturable and stable binding of RNA to GST-HNRNPK but not GST alone. (data not shown). Next, we pre-incubated immobilized GST-HNRNPK or GST with RNA and repeated the PA binding assay. This led to saturable binding of PA to HNRNPK (Figure 5B) with an apparent Kd of 77.3 µM. In addition to saturable binding, concentrations of 50, 100 and 150 μM PA elicited greater response units (RU) when HNRNPK was pre-bound with RNA. Increased RU at lower doses of PA, after RNA binding, seemed inconsistent with the idea that RNA was occluding non-specific binding sites and thus making the interaction saturable. Thus, this observation leads us to favor the interpretation that RNA binding influences the structure of HNRNPK in a manner that creates a specific binding site for PA.

In order to control for the possibility that PA was interacting with RNA rather than RNA-bound HNRNPK, we asked whether PA would bind to a different RNA-binding protein. To this end, we compared PA binding to GST-HNRNPK versus GST-HNRNPI (PTBP1). HNRNPI is a good control since it, like HNRNPK, also binds to pyrimidine-rich tracts. After pre-binding RNA, we found saturable binding of PA to HNRNPK, but not HNRNPI, with an estimated Kd of HNRNPK of 86.2 µM (Figure 5C). This is unlikely to represent the true affinity of PA binding to HNRNPK in vivo because neither the endogenous HNRNPK-binding partners nor the post-translational modifications of native HNRNPK are recapitulated with the bacterial expressed GST-HNRNPK employed in the surface plasmon resonance assay system. In addition, we are able to visualize PA interactions with HNRNPK in HEK293 lysates at concentrations as low as 100 nM of compound 2B (2B TAMRA labeling, data not shown). The lack of interaction between PA and HNRNPI as well as the saturable nature of PA’s interaction with HNRNPK support a specific interaction of PA with HNRNPK.

## 3. Discussion

The marine sponge derivative PA was previously found to have significant neuroprotective qualities in a variety of model systems [6]. Unfortunately, PA does not have the physicochemical properties of a “drug-like” compound; therefore, identification of the target of PA is of significant interest. Using two distinct purification methods to identify candidate targets of PA, followed by a secondary screen for direct interaction, we identified HNRNPK as a target of PA. Follow-up analysis of this interaction by surface plasmon resonance confirmed saturable binding of PA to HNRNPK in an RNA-dependent manner. Our original characterization of PA as being neuroprotective focused heavily on models of motor neuron disease, including Amyotrophic Lateral Sclerosis (ALS). The identification of FUS, TDP-43, and a hexa-nucleotide repeat expansion in the first intron of C9orf72 as causative gene mutations in ALS has put a recent focus on potential dysregulation of RNA processing as a mechanism of neurotoxicity [14,15,16]. For this reason, the fact that PA interacts with an RNA-binding protein involved in many aspects of RNA metabolism is especially intriguing.

HNRNPK is a member of the heterogeneous nuclear ribonucleoprotein (hnRNP) family. These proteins have historically been identified based on their ability to bind heterogeneous nuclear RNA produced by RNA polymerase II as part of the hnRNP complex [17]. Although originally described as part of the hnRNP complex, it is now appreciated that HNRNPK can be found in the cytoplasm and mitochondria, as well as the nucleus [12]. HNRNPK participates in a wide variety of cellular functions including transcriptional control, translational control, RNA transport, splicing, chromatin remodeling and RNA stability [12]. HNRNPK contains three RNA-binding motifs (KH domains) and exhibits robust binding to poly-C regions [18]. In addition to its ability to interact with RNA, HNRNPK interacts with more than 100 protein-binding partners and these reside in the cytoplasm, nucleus and mitochondria [13]. Many of these interactions are thought to be mediated through the K protein interactive (KI) domain, which is a highly unstructured region between KH2 and KH3. HNRNPK is also the target of numerous post-translational modifications including phosphorylation and methylation, which act to integrate numerous signaling pathways [19,20,21].

While identifying a physical target of the neuroprotective compound PA is an important first step, the multitude of HNRNPK-binding partners and functional roles in RNA metabolism represents a significant hurdle to understanding PA’s mechanism of action. Due to this multitude of functions, small molecules that target HNRNPK may have pleiotropic cellular effects. Therefore, rather than design small molecules that target HNRNPK, we favor the use of PA to characterize the specific changes in HNRNPK function that lead to neuroprotection. Future studies aim to use high-throughput sequencing to characterize changes in RNA metabolism after treatment with PA that may mediate PA’s neuroprotective activity. Additionally, these findings suggest that HNRNPK might be of particular importance for further study as a modifier of neurotoxicity in ALS. It has previously been reported that 25 different members of the HNRNP complex interact with the ALS-associated protein TDP-43, and that TDP-43 is also associated with the DROSHA miRNA processing complex, as well as the snRNP complex [22]. The fact that PA is strongly neuroprotective and interacts with HNRNPK suggests that the HNRNP complex and pre-mRNA processing may be of particular interest in mutant TDP-43 mediated neurodegeneration.

## 4. Materials and Methods

**Luciferase Assays:** HEK293 cells were transfected with pGL3-FHRE and pRL-TK plasmids for monitoring FOXO activity or pBIIx-firefly luciferase and pRL-TK for monitoring NFκB activity. Twenty-four hours after transfection, cells were trypsinized and replated into a 96-well plate in drug- or vehicle-treated media, cells were re-dosed at 48 h. Seventy-two hours after drug treatment, the ratio of firefly to renilla luciferase was determined using a Veritas Microplate Luminometer (Promega, Durham, NC, USA) in conjunction with the Dual-Luciferase Reporter Assay Kit (Promega).

**Psammaplysene A and Derivatives:** For synthesis of PA see [1]. PA derivatives used for covalent crosslinking and purification were generated from reaction intermediaries. **Preparation of psammaplysene A-immobilized polymer-coated affinity magnetic beads (FG beads):** Polymer-coated affinity magnetic beads with amine groups (FG beads: [9,23]; TAS8848 N1130 (Tamagawa Seiki Co. Ltd., Iida, Japan)), stocked in pure water, were suspended in ethanol. Prior to immobilization step, ethanol was removed by centrifugation (15,000 rpm, room temperature) of the beads. Then, ethanol, *N*-methylmorpholine in ethanol, PA derivative solution in ethanol, EDC·HCl (1-ethyl-3-(3-dimethylaminopropyl)carbodiimide hydrochloride) solution in ethanol, and 1-hydroxybenztriazole in DMF (*N*,*N*-dimethyformamide) were added to centrifuged beads. After the beads were dispersed well into the medium, the suspension was incubated for 1 day at room temperature. This immobilization step gave Psammaplysene A-immobilized FG beads with unreacted amine groups. Then, the beads were washed with ethanol once and suspended in DMF. Masking of unreacted amine groups on the beads was performed by incubation with 1% acetic anhydride in DMF for 3 h at room temperature. DMF was removed and the PA-FG beads were resuspended in distilled water. Finally, a suspension of the PA-FG beads was kept in pure water at 4 °C.

**PA Target Identification with 2B:** Cell lysates were prepared from HEK293 cells or *C. elegans* in Triton-X 100 buffer (50 mM Tris pH 7.8, 150 mM NaCl, 1% Triton-X 100, complete protease inhibitor cocktail). Lysates were incubated with 250 nM of the PA derivative 2B for 1 h at 4 °C with rotation. The lysate was then UV-illuminated to activate the photo-affinity tag. Crosslinking was performed with 365 nm UV illumination using a Spectroline 6 watt UV lamp on ice. Crosslinked lysates were then processed using the Click-iT protein reaction buffer kit (Molecular Probes, Eugene, OR, USA) to attach either TAMRA–azide or biotin–azide (Molecular Probes) to the alkyene group on 2B. TAMRA-tagged lysates were run on a 4–15% gradient gel and then visualized by in-gel laser scanning with the Typhoon 9400 imaging system (GE Life Sciences, Marlborough, MA, USA). Biotin-tagged lysates were incubated with hydrophilic streptavidin magnetic beads (New England Biolabs, Ipswich, MA, USA) at 4 °C for 1 h with rotation. Beads were washed three times with high stringency wash buffer (PBS with 1 M KCl and 500 mM Urea) to decrease pulldown of indirect binding partners. Beads were submitted for LC–MS/MS analysis to identify binding partners.

**Purification of PA Targets with PA-FG Beads:** HEK293 lysates were incubated with 0X, 1X, and 5X PA-FG beads for 1 h at 4 °C. Beads were then washed 3X in PBS, moved to a new tube and washed 2 additional times in PBS. The beads were then resuspended in 1x SDS-PAGE loading buffer and boiled for 5 min. These samples were then run on a 4–15% gradient gel and visualized with Coomassie Brilliant Blue stain. The bands indicated in Figure 1 were excised and submitted for mass spec.

### ***LC–MS/MS*** ***Analysis***

*In-Gel digestion:* Coomassie Brilliant Blue stained gel bands were excised and cut into 1 mm cubes [23,24]. Briefly, gels were destained with 50% methanol/0.25% acetic acid, reduced with 5 mM DTT (dithiothreitol) (Thermo Fisher Scientific, Waltham, MA, USA), and alkylated with 40 mM iodoacetamide (Sigma, St. Louis, MO, USA). Gels were then washed with 20 mM ammonium bicarbonate (Sigma) then dehydrated with acetonitrile. Trypsin (Promega; 10 ng/µL in 20 mM ammonium bicarbonate) was added to the gel pieces. Proteolysis was allowed to proceed for 4 h at 37 °C [25]. Peptides were extracted with 0.3% triflouroacetic acid, followed by 50% acetonitrile. Extracts were combined and the volume was reduced by vacuum centrifugation.

*In-Solution digestion:* Samples on magnetic beads were washed with 50 mM ammonium bicarbonate. Beads were brought up in 20 mM ammonium bicarbonate, 0.1% Rapigest (Waters, Milford, MA USA) and incubated at 60 °C. Samples were cooled to room temperature, reduced with 5 mM DTT at 50 °C, alkylated with 30 mM iodoacetamide, then digested with 50 ng of trypsin overnight at 37 °C. Samples were acidified with formic acid, incubated at 37 °C for 2 h, spun to pellet cleaved Rapigest and desalted on a C18 ZipTip (Millipore, Billerica, MA, USA). Eluate volume was reduced by vacuum centrifugation.

Tryptic digests were analyzed on a hybrid LTQ OrbitrapXL mass spectrometer (Thermo Fisher Scientific, Waltham, MA, USA) coupled with a 2D NanoLC pump (Eksigent, Dublin, CA, USA) and autosampler. Tryptic peptides were solubilized in 0.1% Trifluoroacetic acid then separated by reverse-phase (RP)–HPLC on a nanocapillary column, 75 µm ID × 20 cm ProteoPep2 (New Objective, Woburn, MA, USA). Mobile phase A consisted of 1% methanol, 0.1% formic acid and mobile phase B of 1% methanol, 0.1% formic acid, 80% acetonitrile. Peptides were eluted into the mass spectrometer at 300 nL/min with each RP–LC run comprising a 15 min sample load at 3% B and a 90 min linear gradient from 5 to 45% B. The mass spectrometer was set to repetitively scan *m/z* from 300 to 1800 (R = 100,000 for LTQ-Orbitrap) followed by data-dependent MS/MS scans on the ten most abundant ions, with a minimum signal of 1500, dynamic exclusion with a repeat count of 2, repeat duration of 15 s, exclusion size of 500 and duration of 60 s, isolation width of 2.0, normalized collision energy of 28, and waveform injection and dynamic exclusion enabled. Fourier Transform Mass Spectromemtry (FTMS) full-scan AGC target value and MSn AGC were 1 × 10^6^ and 5 × 10^3^, respectively. FTMS full-scan maximum fill time was 500 ms, while ion trap MSn fill time was 50 ms; microscans were set at one. FT preview mode; charge state screening, and monoisotopic precursor selection were all enabled with rejection of unassigned and 1+ charge states.

All MS/MS data were analyzed using Mascot (version 2.3.02, Matrix Scientific, Elgin, SC, USA). Mascot was set up to search a human subset of the Uniprot Swissprot/Tremble protein sequence database (83,908 entries) assuming the digestion enzyme semiTrypsin. The database search was performed with a fragment ion mass tolerance of 0.80 Da and a parent ion tolerance of 50 ppm. Iodoacetamide derivative of cysteine was specified in Mascot as a fixed modification. Oxidation of methionine was specified in Mascot as a variable modification. Scaffold (version Scaffold_3_00_08, Proteome Software Inc., Portland, OR, USA) was used to validate MS/MS-based peptide and protein identifications using the Peptide Prophet algorithm [26] to establish a 1% false discovery rate.

**Surface Plasmon Resonance:** PA binding to GST-HNRNPK was analyzed using a CM5 sensor chip on a BIACORE 3000. Anti GST antibody was immobilized on the sensor chip using the GST Capture Kit (GE). A capture/crosslink strategy was used to prevent dissociation of GST and GST fusions from the GST antibody over time. The anti GST antibody was saturated with GST or GST fusion protein and then briefly pulsed (60 s) with NHS/EDC (*N*-hydroxysuccinimide/1-ethyl-3-(3-dimethylaminopropyl)carbodiimide) followed by a brief pulse (60 s) of ethanolamine. The buffer used for dissolving PA and washing the surface was composed of 5% DMSO and 0.05% Tween-20 in PBS. The binding space was explored in an iterative fashion over a broad range of concentrations until saturable binding conditions were achieved. PA was injected at a flow rate of 30 μL per minute for 60 s followed by a 120 s wash before administration of the next drug concentration.

## Figures and Tables

**Table 1:** Gene Ontology Analysis of PA-Interacting Complex.

**Biotinylated 2B Pulldown**							
**Term**	**Count**	**%**	**List Total**	**Pop Hits**	**Pop Total**	**Fold Enrichment**	**FDR**
GO: 0016071~mRNA metabolic process	14	45.16	31	370	13,528	16.51	4.04 × 10^−10^
GO: 0008380~RNA splicing	13	41.94	31	284	13,528	19.98	4.74 × 10^−10^
GO: 0006397~mRNA processing	13	41.94	31	321	13,528	17.67	2.02 × 10^−9^
GO: 0000377~RNA splicing, via transesterification reactions with bulged adenosine as nucleophile	10	32.26	31	153	13,528	28.52	3.68 × 10^−8^
GO: 0000398~nuclear mRNA splicing, via spliceosome	10	32.26	31	153	13,528	28.52	3.68 × 10^−8^
GO: 0000375~RNA splicing, via transesterification reactions	10	32.26	31	153	13,528	28.52	3.68 × 10^−8^
GO: 0006396~RNA processing	14	45.16	31	547	13,528	11.17	5.63 × 10^−8^
**PA-FG Bead Pulldown**							
**Term**	**Count**	**%**	**List Total**	**Pop Hits**	**Pop Total**	**Fold Enrichment**	**FDR**
GO: 0006396~RNA processing	43	27.22	132	547	13,528	8.06	1.23 × 10^−23^
GO: 0016071~mRNA metabolic process	36	22.78	132	370	13,528	9.97	4.51 × 10^−22^
GO: 0006397~mRNA processing	33	20.89	132	321	13,528	10.54	1.35 × 10^−20^
GO: 0008380~RNA splicing	31	19.62	132	284	13,528	11.19	7.54 × 10^−20^
GO: 0000375~RNA splicing, via transesterification reactions	22	13.92	132	153	13,528	14.74	1.78 × 10^−15^
GO: 0000398~nuclear mRNA splicing, via spliceosome	22	13.92	132	153	13,528	14.74	1.78 × 10^−15^
GO: 0000377~RNA splicing, via transesterification reactions with bulged adenosine as nucleophile	22	13.92	132	153	13528	14.74	1.78 × 10^−15^
GO: 0010608~posttranscriptional regulation of gene expression	14	8.86	132	211	13,528	6.80	0.000203
GO: 0043489~RNA stabilization	6	3.80	132	15	13,528	40.99	0.000345
GO: 0048255~mRNA stabilization	6	3.80	132	15	13,528	40.99	0.000345
GO: 0043488~regulation of mRNA stability	6	3.80	132	22	13,528	27.95	0.002864
GO: 0043487~regulation of RNA stability	6	3.80	132	24	13,528	25.62	0.004552

**Gene ontology analysis of candidate proteins.** The candidate PA-interacting proteins identified by each approach were used as an input for gene ontology analysis, and analyzed for enrichment in function as compared to the entire proteome. Terms with an False Discovery Rate (FDR) < 0.01 are displayed. Both purification approaches yielded a robust enrichment in proteins involved in RNA processing, suggesting that PA’s neuroprotective activities might be mediated through changes in RNA metabolism.

## Supplementary Materials

The following are available online at www.mdpi.com/1660-3397/15/8/246/s1. Excel file: S1_Samples_Report.xlsx, Complete mass spec data for the 2B and PA-FG purifications. Figure S1: Cytoplasmic vs. nuclear distribution of HNRNPK in HEK293 cells.
