# Supplementary figures and images for: The Neuroprotective Marine Compound Psammaplysene A Binds the RNA-Binding Protein HNRNPK
Source: Mar Drugs. 2017 Aug 7;15(8):246. doi: 10.3390/md15080246 (PMC5577601; doi:10.3390/md15080246)

# Supplemental figure 1.

cyto

nuclear

IB: hnRNP K

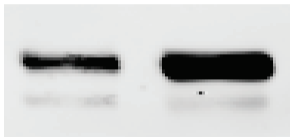

IB: Actin

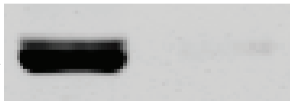

Supplement: Supplementary file 1 [file marinedrugs-15-00246-s001.zip › marinedrugs-211484-supplementary.pdf]
